# A qualitative study trialling the acceptability of new hepatitis C prevention messages for people who inject drugs: symbiotic messages, pleasure and conditional interpretations

**DOI:** 10.1186/s12954-015-0042-5

**Published:** 2015-03-04

**Authors:** Carla Treloar, Jamee Newland, Louise Maher

**Affiliations:** Centre for Social Research in Health, UNSW Australia, Sydney, NSW Australia; Population Health Services, Nepean Blue Mountains Local Health District, Penrith, NSW Australia

**Keywords:** Hepatitis C, Prevention, Education, People who inject drugs, Needle and syringe programs

## Abstract

**Aim:**

Prevention of hepatitis C (HCV) remains a public health challenge. A new body of work is emerging seeking to explore and exploit “symbiotic goals” of people who inject drugs (PWID). That is, strategies used by PWID to achieve other goals may be doubly useful in facilitating the same behaviours (use of sterile injecting equipment) required to prevent HCV. This project developed and trialled new HCV prevention messages based on the notion of symbiotic messages.

**Method:**

New HCV prevention messages were developed in a series of 12 posters after consultation with staff from needle and syringe programs (NSPs) and a drug user organisation. Two posters were displayed each week for a 6-week period within one NSP. NSP staff and clients were invited to focus groups to discuss their responses to the posters.

**Results:**

A total of four focus groups were conducted; one group of seven staff members and three groups of clients with a total of 21 participants. Responses to each of the posters were mixed. Staff and clients interpreted messages in literal ways rather than as dependent on context, with staff concerned that not all HCV prevention information was included in any one message; while clients felt that some messages were misleading in relation to the expectations of pleasure. Clients appreciated the efforts to use bright imagery and messages that included acknowledgement of pleasure. Clients were not aware of some harm reduction information contained in the messages (such as “shoot to the heart”), and this generated potential for misunderstanding of the intended message. Clients felt that any message provided by the NSP could be trusted and did not require visible endorsement by health departments.

**Conclusions:**

While the logic of symbiotic messages is appealing, it is challenging to produce eye-catching, brief messages that provide sufficient information to cover the breadth of HCV prevention. Incorporation of symbiotic messages in conversations or activities between staff and clients may provide opportunities for these messages to be related to the clients’ needs and priorities and for staff to provide HCV prevention information in accord with their professional ethos.

**Electronic supplementary material:**

The online version of this article (doi:10.1186/s12954-015-0042-5) contains supplementary material, which is available to authorized users.

## Background

Hepatitis C remains a significant public health challenge. Australian estimates show that almost 10,000 new infections occur each year, with a total of about 250,000 Australians living with chronic hepatitis C infection [1]. Approximately 90% of new infections occur among people with a history of injecting drug use [2]. Prevention of hepatitis C has relied on the availability of sterile injecting equipment through public and private outlets, as well as education conducted by health authorities and non-government agencies [3].

In Australia, prevention of hepatitis C among people who have injected drugs (PWID) has not been as successful as efforts to prevent HIV. Australia enjoys a very low prevalence of HIV among PWID [4], and this success is associated with the early introduction of needle and syringe programs (NSPs) [5] and the changes in behaviour made by PWID in response to prevention messages. While the effectiveness of NSPs in HIV prevention has been documented as one of the world’s most significant health interventions [6,7], the impact of NSP in reducing hepatitis C transmission is more contested [8].

These structural interventions and community responses have not been sufficient to achieve similar results in hepatitis C. This is thought to be the result of the higher background prevalence of hepatitis C within the population at the time of introduction of NSPs and a higher rate of infectivity of hepatitis C than HIV [9]. Further, there is limited evidence to evaluate the coverage of NSP services needed to achieve optimal saturation of sterile equipment [10-12]. Besides the distribution of sterile injecting equipment, NSP staff also typically engage in face-to-face encounters with PWID clients and have opportunity to provide or direct clients to health promotion messages. Despite high level support for the use of information, education and communication to prevent hepatitis C [13], there is little literature available on the best ways to develop and subsequently disseminate optimal health promotion messages. While improvements and innovation in NSP programming continue, for example, via vending machine, and in collaboration with primary health care [14-17], there has been little innovation or evolution in hepatitis C prevention messaging. A recent review of print-based hepatitis C resources showed that messages tended to replicate previous messages with only incremental change [18]. Although some evolution of messages had occurred and there were some limited instances of innovative packaging and tailoring of messages to address specific sub-groups, the overall finding of the review suggested that current prevention messages addressed PWID without distinction in terms of site or setting of drug use, gender, age, regional location or other personal, social or structural factors that are known to influence injecting practice and hepatitis C transmission risk.

At the same time, a new body of work is emerging which seeks to explore and exploit “symbiotic goals” of PWID [19]. That is, strategies used by PWID to achieve other goals may be doubly useful in facilitating the same behaviours (use of sterile injecting equipment) required to prevent hepatitis C [20]. Research conducted in New York, Sydney and London has documented the following strategies as simultaneously promoting use of sterile injecting equipment: avoiding drug withdrawal; finding ways to manage withdrawal; maintaining social support from nonuser family and friends; socially embedding protective norms among networks of PWID; avoiding injecting related scars or marks; maintaining venous access; “going under the radar” to pass in society; using at home or in private [21-23]. These “symbiotic” messages are also useful to consider in the context of “hepatitis C prevention fatigue” amongst the target group, where over-exposure to hepatitis C prevention messages may result in little attention being paid to such messages as they are perceived to contain no new information [24,25].

In the USA, a formal education program has been developed from these findings to engage PWID in a 1-week, five-session intervention. The pilot of this intervention has shown promising results in relation to reduced drug intake, injection-related risk behaviour, drug withdrawal and risky injecting networks as well as increases on factors such as planning skills, self-efficacy and stigma management [26].

In the context of brief interventions between NSP staff and clients, a formal intervention program is not feasible. Such interventions are typically initiated by staff and draw on existing resources within the NSP service, such as printed health promotion materials. Incorporating symbiotic messages into interactions between NSP staff and clients may be a means to develop a new generation of messages that encourages innovation in a sector within which current messages are perceived as stale and may therefore have less than optimal effects in relation to intended impact. However, we do not know how messages that do not explicitly mention hepatitis C or messages that may privilege notions of pleasure associated with drug use will be perceived or interpreted by health workers or PWID.

The aim of this pilot study was to examine the acceptability of such messages and the feasibility of implementing these messages in the NSP environment. Within this project, health promotion materials which used “symbiotic messages” for hepatitis C prevention were produced and displayed in the NSP. The acceptability of messages and the feasibility (from the perspective of NSP staff and clients) of including these in the repertoire of prevention messages used in the NSP setting were examined using qualitative methods.

## Method

In Australia, NSPs are typically publicly funded services that operate in a range of ways—primary (purpose-built, stand-alone services with specialist NSP staff), secondary (injecting equipment is distributed within other health services such as community health or sexual health services) or vending machine. Privately owned pharmacies may also provide equipment for sale or free on exchange. This study was conducted in a primary NSP staffed by a range of health workers (such as those with qualifications in health education/promotion, nursing or social work) located in Sydney, New South Wales (NSW) and servicing on average 30 clients per day and distributing approximately 260,000 needle/syringe units per year across the counter. The health promotion messages were produced in poster form and displayed on the walls of the NSP. Two posters were displayed per week for a total of 6 weeks. A diary was provided in which staff were asked to record clients’ comments and reactions to the posters. The diary entries were not used in analysis.

Three focus groups were conducted with NSP clients. A flyer was placed in the NSP and staff alerted clients to this. Participants were required to be aged 18 or above and have sufficient English language skills to consent and engage in a focus group discussion. Discussion topics covered in all client groups included the following: previous messages that have attracted participants’ attention and influenced their knowledge or practice; awareness of and reactions to the messages (including in relation to previous messages); influence of the messages on injecting practices; extent of discussion about the messages with other people. Clients were reimbursed with $20 to acknowledge their contribution, and light refreshment was also provided.

One focus group was conducted with NSP staff who were recruited using a letter of invitation sent via their manager. Discussion topics included the following: reactions of clients to the posters; staff reactions to each poster (posters discussed individually); and, what would need to happen if these messages were to become permanent parts of NSP health promotion?

The discussion was digitally recorded and transcribed verbatim by professional transcription services. The audio recording of client focus group #1 was inaudible and the researcher produced detailed notes following the group including verbatim quotes where possible. All project data were entered into NVivo qualitative data analysis software (V10). Using both deductive and inductive analytic processes [27], data was examined to describe reactions to each of the 12 posters with respect to the appropriateness of the message, the imagery and suggestions for improvement. The authors then met to discuss themes emerging across the set of posters to produce understandings or principles that would guide revision of posters including how these could be incorporated into everyday practice of NSP staff and meet organisational expectations. This study was conducted in compliance with the Helsinki Declaration and had approval from the human research ethics committee of the relevant health authority.

### Design and content of posters

The posters were a combination of existing and newly developed messages. The existing posters had been developed by the Irish Needle Exchange Forum (Additional files 1, 2, 3 and 4) upon review of the emerging literature regarding symbiotic messages. These existing posters were re-produced to show local injecting equipment. The new messages (Additional files 5, 6, 7, 8, 9, 10, 11 and 12) were developed specifically for this project (by LM) and informed by the results of consultation with NSP staff and with staff of a drug user organisation.

None of the posters specifically mentioned hepatitis C prevention. The symbiotic goals included in the posters were prevention of track marks (Additional files 5 and 7), maintenance of venous access (use of sterile equipment, rotating injecting sites, drinking water prior to injection, injecting with the flow of blood that is, “to the heart”—Additional files 4, 5, 7, 8, 10, 12), prevention of pain associated with injecting (Additional files 2 and 3) and advice on planning in relation to equipment, location, money and time (“a great hit is all in the planning”, “prior planning prevents problems”—Additional files 6, 9).

The messages also included direct reference to pleasure of drug use in a positive framing (“blow your brains not your veins, use a new fit for every hit”—Additional files 1 and 10) with mixed positive and negative frames (“there’s a fine line between pleasure and pain, so use a pick once don’t use it again”, “new fit for a perfect shot, ’cause blunt fits blows veins”—Additional files 11 and 12) and in a negative frame (“re-using fits hurts, new fit every hit”, “ouch…that hurts!! Use a new needle and say goodbye to ouch”—Additional files 2 and 3).

## Results

A total of 21 clients participated in three focus groups (four to eight participants per group) with an average age of 43.7 years (range 29–60 years) and included ten males. All client participants had an extensive history of injecting (>10 years), and most reported injecting heroin but poly-drug use was common. Seven staff participated in the discussion.

### Responses to symbiotic messages: absence of hepatitis C and useful information

The messages were positively received by clients and staff in relation to providing information that was useful to clients and for drawing attention to issues within the lives of clients that were of priority to them, rather than reiterating well-worn hepatitis C prevention messages. In addition, some messages provided information that was previously unknown, magnifying participants’ evaluation of the interest and usefulness of these messages.

The fact that there was no mention of hepatitis C in these posters was not explicitly noted by client participants until it was raised in focus group discussions by the researcher. When the omission of hepatitis C was brought to the attention of clients who provided feedback on the posters at the NSP, a staff member explained that the clients understood and were not interested in further repetition of standard hepatitis C messages.

did appreciate it and they understood that all these things are necessary and we are not still banging on about hepatitis C—“if you do this then you won’t get hepatitis C”. So they understood the rationale behind it (Staff group).

The messages are strong on their own, I mean I think [the other staff members’] comment before about not realizing there is no hepatitis C is because the message is implicitly there without, you don’t need to mention hepatitis C. That’s kind of my way of seeing it, the strength of the posters, is that they look nice, they draw you in without ramming hepatitis C messages down your throat … Because [clients] do get sick of [hepatitis C] (Staff group).

Client participants explained that the omission of hepatitis C in the posters was important for attracting a wider audience as other issues were of higher priority for PWID: “I think without it you will get a broader market” (Client group #3). Additionally, hepatitis C appeared to be of lower priority and not perceived as serious as other issues facing PWID: “hepatitis C is not a priority anyway, for us it’s about making sure you have a vein” (Client group #1).

These symbiotic messages contained new information for some clients or information that clients with a longer history of injecting wished that they had been aware of when they first started injecting, such as information about drinking water to make injection easier and the direction of injection (towards the heart).

Well because those three were like three subjects [Additional file 8] you’ve got there they’re really important, you know what I mean, a new fit, that goes without saying, shoot to the heart, I never knew that until quite a few years ago and now I realize how important it is and the water, that’s another one, I go for a blood test and I have got to drink at least 2 litres of water before I can go and get my blood tested so that my veins are. (Client group #3).

However, the message regarding “shoot to the heart” (Additional files 7 and 8) was somewhat controversial in that it assumed knowledge among the client group and was therefore positioned as a reminder, rather than explaining what this instruction meant in practice. The lack of understanding about direction of injection was explained as relevant both to younger and to more experienced PWID.

They would probably try to stick the fit *in* their heart: especially if they’ve seen [the movie] Pulp Fiction (Client group #3).

You could take that [message regarding “shoot to the heart”] anywhere and explain to a lot of people what that means because I tell you there’s a lot of people that wouldn’t understand that. I have been using for a long fucking time and I didn’t quite understand it: Yeah, I’ve been going for 20 years or something (Client group #2).

### Non-conditional interpretations of symbiotic messages

There was also some negative reaction among staff and client participants to the use of symbiotic messages based on literal or non-conditional interpretations of these messages. Participants examined and interpreted the content of the poster which was intentionally brief and contained limited information. What was missing from the message was questioned, primarily by staff (as well as a minority of clients), particularly in relation to the absence of complete information about hepatitis C prevention. That is, the poster images and messages were criticised for the inclusion of only the needle and syringe and not the other equipment required for the best chance of preventing transmission of hepatitis C. In this way, participants interpreted the messages in absolute or literal terms, as though it should provide the full description of hepatitis C prevention.

I’m not happy with the message [Additional file 8]. I don’t think that the 3 top tips chosen are the most important or exclusive to giving a great hit. I would have chosen other things, such as sterile water. Drinking plenty of water is important to your health but injecting requires all equipment to be bug free to stay healthier and make a ‘great hit’ not a ‘bad hit’. (Staff focus-group).

Because they are giving you false information: you are spruiking about all this ‘not using somebody else’s fit’ or making sure that ‘you use a new fit every hit’. If you use a new fit every time, there’s still no guarantee that you are not going to get hep C because you can get it from the water, you can get it from the spoon, you can get it from any other piece of equipment that somebody else has used. You use on your own, with your own equipment, all the time and that’s the only way that you can guarantee that you are not going to get it (Client group #2).

In a similar way, client participants were critical of the ways in which the messages implied that a pleasurable experience could be achieved by following the advice contained in the posters. The posters indicated that using new equipment or planning for injection could result in “great” or “better” experiences (such as Additional files 1, 8, 9 and 12). Clients interpreted these messages literally or in absolute terms. That is, clients drew on their experience of injecting drug use to compare the limited range of factors presented in the posters with the broader range of factors that could influence the experience of drug use. These comments indicated that client participants felt the messages were misleading in that the use of sterile equipment alone cannot guarantee a “better” experience.

Yeah I don’t really like it because it leads you to believe that if you do use a new fit you’ll have better shot and it totally misleads you. [*Researcher: In what way?*] Well, in a way that people think oh well if I go and get a new fit I might get a better hit, a better high … Well ‘better’ to me means yes there are options because I can go and get a better car than him, I have the option or I can get a better pair of pants than [him] or a better necklace than [her] (Client group #2).

I believe it is giving the wrong idea. That if you plan and plan prior, you won’t have any problems, you know what I mean but we can’t prevent the police coming and knocking at the door (Client group #2).

Or your dealer taking three hours or your mate taking five. So you go where are you [and then] well that isn’t what you said half an hour ago. Yeah right, I’m 10 minutes away and 10 minutes turns into two hours (Client group #2).

### Modes of presentation

#### Imagery and colour

The posters were designed to deliberately move away from traditional and authoritative modes of presentation to include bright imagery, vivid colours, use of humour and colloquial language. The use of bright, eye-catching colours and images was appreciated as clients reported that the NSP environment contains a number of posters and that these compete for attention. For example, poster #4 (grey background) was noted as “dark and drab”, “very grim”, “dull” and dirty” by staff and that “there’s nothing to draw your attention to that, if it was amongst a whole heap of other things you wouldn’t look at that one” (Client group #2). Other posters (such as Additional files 10, 11 and 12) were noted as bright, attention drawing and “fun” in their use of colour.

A light-hearted or colourful approach to these messages was not endorsed by all participants. There were calls among client participants for more confronting or shocking images that portrayed track marks in vivid detail as it was suggested that this imagery may attract more attention than standard messages which are very familiar and hence less visible to NSP clients.

I am more into confronting things; things need to be more confronting.… it’s got to be more full on, it really does, that’s my opinion … Hey if you want confronting pictures, I am more than happy for you to fucking pincushion me and just don’t show my face. Well to be honest no I don’t think there is enough [confronting messages being developed]. I don’t know if I have been blinded but I have not really noticed much (Client group #2).

These persons they are not like proper users or something, if you are talking about track marks you should have somebody that’s got trackies all over them, like I’ve seen some bad trackies, it’s like all over their arms (Client group #3).

Across a number of posters, participants noted a “disconnect” between the imagery and the message. In particular, any poster displaying needles/syringes that were uncapped (such as Additional file 3) immediately drew responses that related to inappropriate discarding of used equipment, rather than the intended message of the poster.

Dumped syringes, which were elicited by picture of a needle on the grass are an example. No mention was made of discarded needles [in the posters] but it was what most people talked about (Staff group).

### Humour and colloquial language

The use of humour and colloquial language was divisive, was perceived as potentially reinforcing stereotypes and was leading to possible misinterpretation. The poster “Blow your brains, not your veins … use a new fit for every hit” (Additional file 10) was designed to emphasise the pleasure of drug use and the importance of sterile equipment to maintain venous access. However, the term “blow your brains” provoked some confusion among participants as the message could be interpreted as “inciting to overdose or episode or something—it’s like yeah and then go psychotic as well, that would be great, yeah, blow your brains and then go psycho” (Staff group). Clients also noted that the wording “make us sound illiterate” (Client group #2).

The poster “these are the only trackies you want to be seen in” (Additional file 7) generated the most divisive responses. The term “trackies” was intended as a humorous play on words referring both to track suit pants as well as track marks as a result of injection. There were staff and client participants who noted this as “clever”, “hilarious” and “a good play on words”. However, the potential for the track suit pant imagery to mark NSP clients as both drug users and from a lower socio-economic area of Sydney was noted by staff and clients.

I think it is brilliant but clients hated it. [They said] you wouldn’t fucking put that up [in a higher socioeconomic area] would you, why do you think we are … dags? Yeah, they’re terrible trackies and thongs (Staff group).

I don’t think there is anything stylish or whatever about wearing trackies.

yeah, it stigmatizes it looks like they cost $5 or $10 in the shop; he may as well be wearing the hoodie. [*looking around the room to comment on what participant were wearing*] like you’ve got a Zoo York shirt on, I’ve got a Ralph Lauren, you know what I mean, we like to present (Client group #2).

### The absence and perils of pleasure

Previously, we have noted reactions from client participants calling for shocking and confronting messages rather than messages that referenced the pleasurable aspects of drug use. Besides this, there was no discussion of pleasure among client participants. Staff participants endorsed the inclusion of pleasure-based messages but indicated the unwanted attention and increased vulnerability of NSP services that could be generated by wider dissemination of such messages, including that these messages “would be picked up and slammed outside of an NSP” (Staff group).

### Authority and legitimacy of these health promotion posters

Given the potential sensitivity of these posters, they were displayed without any formal signifier of where or by whom they had been developed. Whether these posters carried the same authority as health promotion materials which carried institutional imprimatur (such as from the relevant health authority) was explored with participants. One staff participant explained that they believed “the posters gained authority simply by being well produced and displayed in a hospital space” (Staff group). However, client participants identified that their perceptions of the posters’ authority was not created because the poster was located within a hospital space; rather that their perceptions of these posters was linked to the trusting relationships that clients had with the NSP staff. Clients explained that the NSP staff were respected and valued and therefore there would be more engagement with information that was displayed in this environment. While some staff members believed that “the use of a [hospital] logo, [would] endorse the message and its legitimacy”, the inclusion of a logo from a health authority appeared to be not particularly relevant for NSP clients when making assessments about message authority.

Lacks authority? Whose authority? [*Researcher: For example the health department or the hospital management*]. Well what sort of authority do they think they have anyway? (Client group #1).

## Discussion

No individual poster received full endorsement by either NSP staff or client participants revealing the difficulty of producing messages that are attractive to and resonate with a diverse audience. Moving away from traditional modes of presenting health promotion messaging to use pleasure and symbiotic messages was also not universally perceived as acceptable by either staff or client participants. As such, the development of symbiotic messages requires both some revision of the messages, as well as further thought on how NSP clients and staff can be supported to engage with messages that are unusual and currently not acceptable to them.

Symbiotic messages were recognised as appealing, in theory, for NSP client participants. It was acknowledged that PWID prioritise other aspects of their lives over hepatitis C and that this provided the logic upon which to build messages that speak to these priorities. However, NSP staff participants expressed strong reservations about the limitations of these messages in not providing the full range of information about ways in which to prevent hepatitis C. In this way, we can see a mismatch (or disconnect) between the focus of the poster and the professional ethos of the NSP worker. Client participants also suggested these posters may be providing a false sell because the message implies the promise of a better or perfect injecting experience on the basis of only a very limited set of parameters. Both staff and client accounts can be viewed in terms of a literal or “absolute” rather than “conditional” (as relevant to and dependent on context) interpretation of these messages [28]. These messages were not intended to express that their content held the secret of untold pleasure or complete viral management; but in the absence of other opportunities to moderate or augment these messages, this is how they were perceived by some participants. Taking the endorsement of symbiotic messages together with the “absolute” nature of their interpretation suggests that it is necessary to find ways to moderate this interpretation and introduce a “conditional” framing that provides opportunity for those messages to be discussed by clients and applied to their own needs and priorities.

These data suggest that hepatitis C health promotion efforts that use symbiotic messages may be best conceived of ways to facilitate additional interactions between staff and clients in which staff are able to piggy-back the full range of hepatitis C prevention messages and clients can examine this information in the context of their own experience and circumstances. These interactions may be assisted by activities that help to cement the clients’ awareness of the messages. For example, when messages displaying the importance of water for vein health are displayed, this could be accompanied by the provision of a water fountain or reusable water bottles to augment the written message. Given the lack of awareness about the importance of injection direction, posters displaying the “shoot to the heart” message could be accompanied by activities in which clients are engaged with physical props which indicate the appropriate direction to inject in various parts of the body. Messages about the pleasure of injecting (“better hit” or “perfect hit”) could be accompanied by an interactive exercise in which clients note what influences their experiences. In these ways, NSP staff are also demonstrating their understanding of client’s experiences and moving interactions to topics broader than hepatitis C but which can encompass hepatitis C prevention messages.

The symbiotic message literature has raised a number of potential factors on which to build intervention messages. The posters designed and trialled in this study focused on a number of these factors. Other messages could be developed and trialled around factors such as maintaining social relationships with people with resources, avoiding withdrawal, maintaining a reliable source of income and social norm processes such as embedding safety within the injecting network. Developing these messages should also focus on clear target groups for each message.

Although staff can demonstrate awareness of client’s needs, the local community may be less aware of or empathic towards the experience of clients and the ways in which NSP services can best address their needs. The low tolerance of services for PWID among the community, media and political spheres has been well established [29-32]. Therefore, the use of health education materials that denote pleasure in relation to illicit drug use potentially brings additional vulnerability to the NSP sector. Building on these posters to produce a range of activities for NSP staff and clients also firmly designates these posters as “within” NSP resources, that is, not expected to be removed from the NSP. This alleviates some of the potential sensitivity about pleasure-based messages in relation to an illegal activity and retains messages within the NSP while reinforcing that these messages are not appropriate for use in take-away materials such as pamphlets.

## Conclusions

These new messages and activities may change the ways in which NSP staff and clients have previously interacted. These messages have the potential to provide space for NSP staff to speak with clients about other issues and to introduce activity into the interaction, thereby deepening the level of engagement. In NSW where this study was conducted, the NSP guidelines specify the main goals of NSP activity as the prevention of blood-borne viruses with a secondary objective of providing referrals to other services [33]. There is no recognition of the ways in which NSPs can directly act on the wider sets of needs with which PWID may present [16]. In this environment, NSP staff may feel “locked” into a certain mode of practice, with hepatitis C prevention as a priority that shapes their approach to each educational interaction with clients. These results demonstrate that (i) NSP clients trust NSP staff to present them with legitimate and worthwhile information which is relevant to their needs, (ii) NSP clients endorse the notion of symbiotic messages as a means to attract and engage audiences who may otherwise ignore the plethora of health messages with which they are confronted, and (iii) that there are ways in which to incorporate a focus on “symbiotic messages” to meet the professional ethos of NSP staff. Although this research drew on practitioners’ use of previous research results (via the Irish Needle Exchange Forum), further work is required to ensure that all aspects of the research are trialled and that advocacy is directed towards policy makers and managers of NSP services to keep them informed of emerging insights into innovative ways to provide hepatitis C prevention within the NSP setting which, on face value, go beyond the narrow focus of hepatitis C prevention but which may work to achieve that goal.

## Additional files

Additional file 1:
**Poster 1—New fit … better hit.**
Additional file 2:
**Poster 2—OUCH … that hurts!!.**
Additional file 3:
**Poster 3—Re-using fits hurts.**
Additional file 4:
**Poster 4—Save your vein for next time.**
Additional file 5:
**Poster 5—Some things are meant to be seen.**
Additional file 6:
**Poster 6—Prior Planning Prevents Problems.**
Additional file 7:
**Poster 7—These are the only trackies you want to be seen in.**
Additional file 8:
**Poster 8—3 top tips for a great hit.**
Additional file 9:
**Poster 9—A great hit is all in the planning.**
Additional file 10:
**Poster 10—Blow your brains … not your veins.**
Additional file 11:
**Poster 11—It’s a fine line between pleasure and pain.**
Additional file 12:
**Poster 12—New fit for a perfect shot—bullseye.**
