# Supplementary figures and images for: A qualitative study trialling the acceptability of new hepatitis C prevention messages for people who inject drugs: symbiotic messages, pleasure and conditional interpretations
Source: Harm Reduct J. 2015 Mar 4;12:5. doi: 10.1186/s12954-015-0042-5 (PMC4355982; doi:10.1186/s12954-015-0042-5)

# NEW fit... better HIT

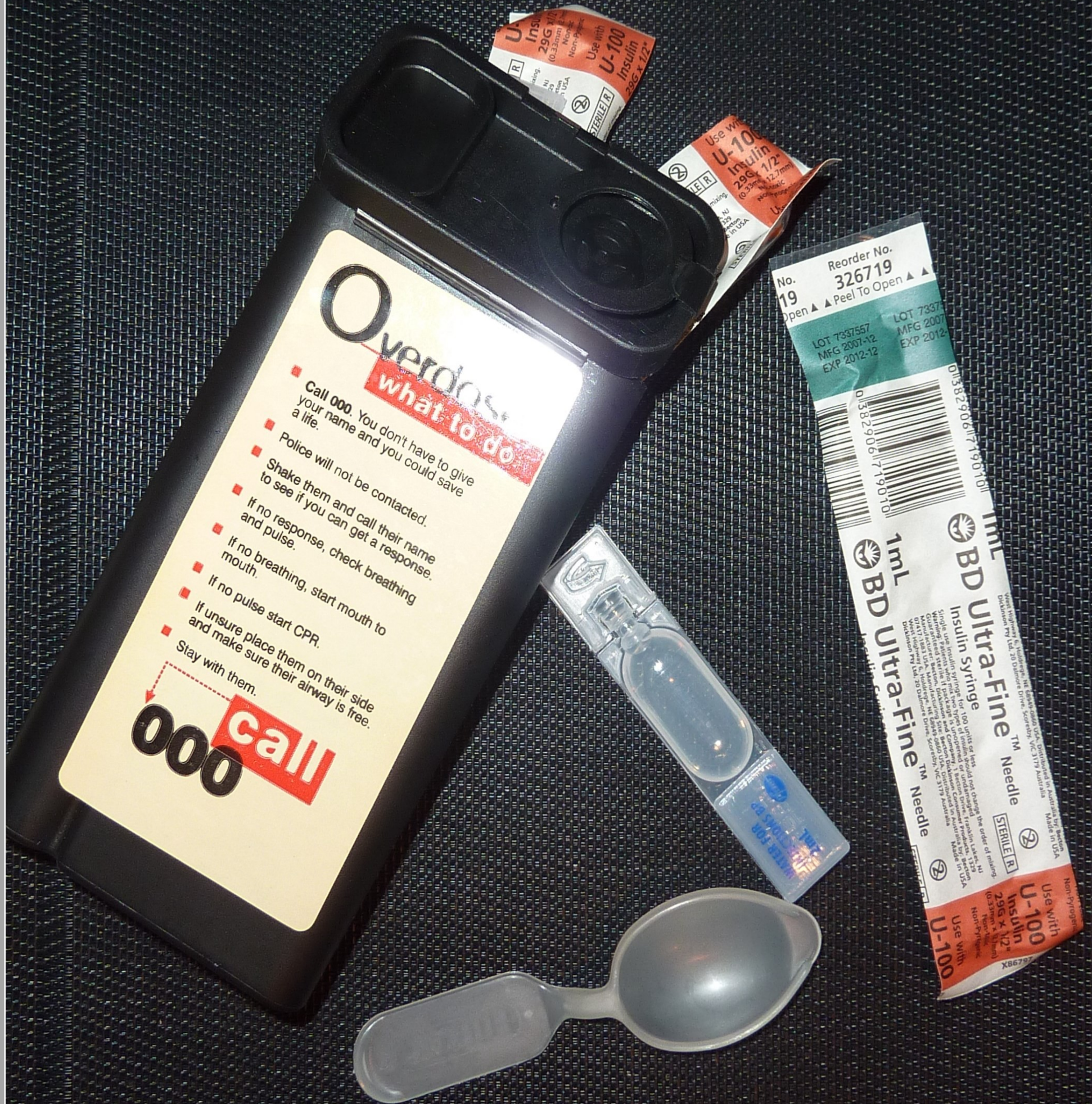

Supplement: Additional file 1: — Poster 1—New fit … better hit. [file 12954_2015_42_MOESM1_ESM.pdf]

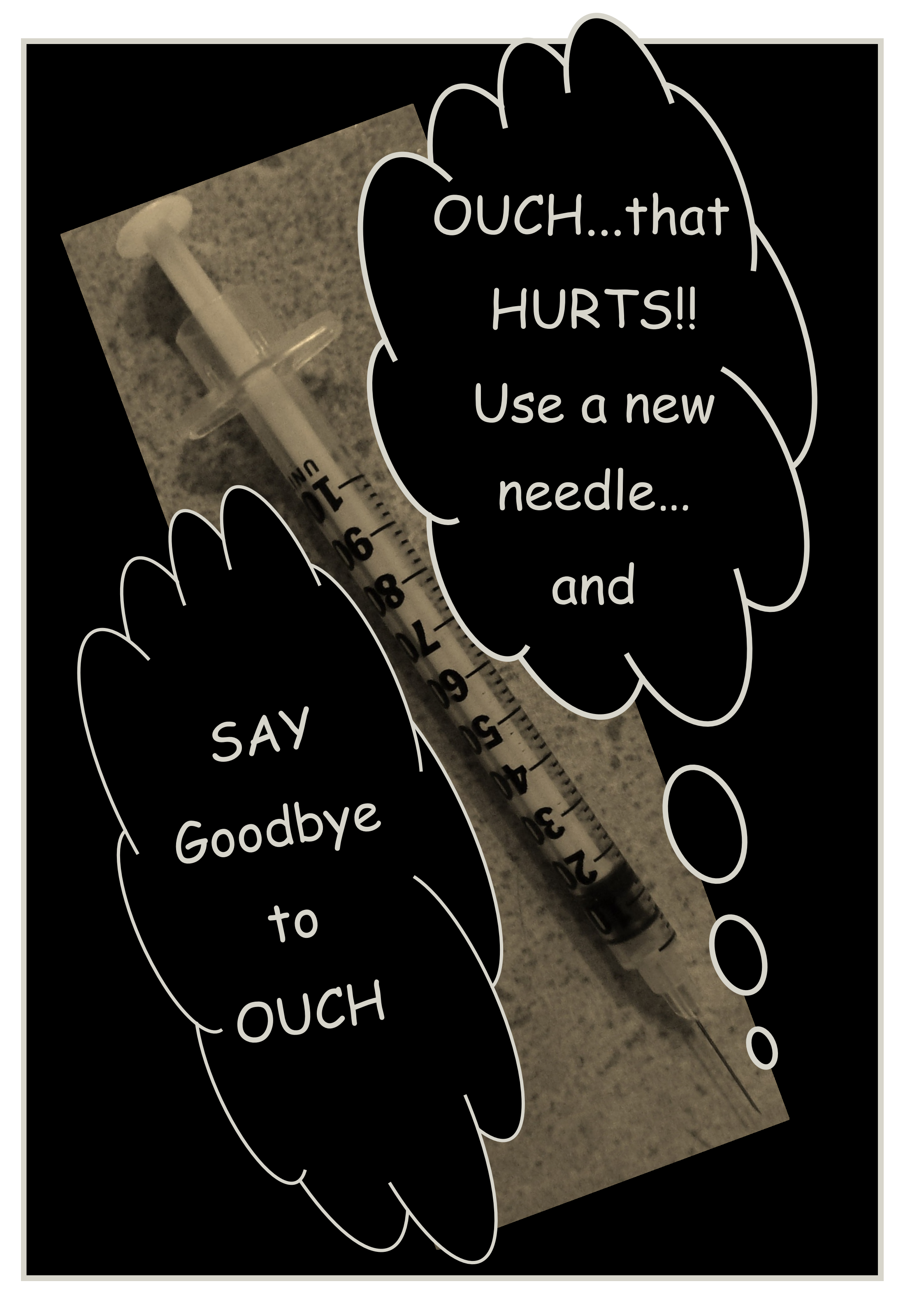

OUCH...that  
HURTS!!

Use a new  
needle...  
and

SAY  
Goodbye  
to  
OUCH

Supplement: Additional file 2: — Poster 2—OUCH … that hurts!!. [file 12954_2015_42_MOESM2_ESM.pdf]

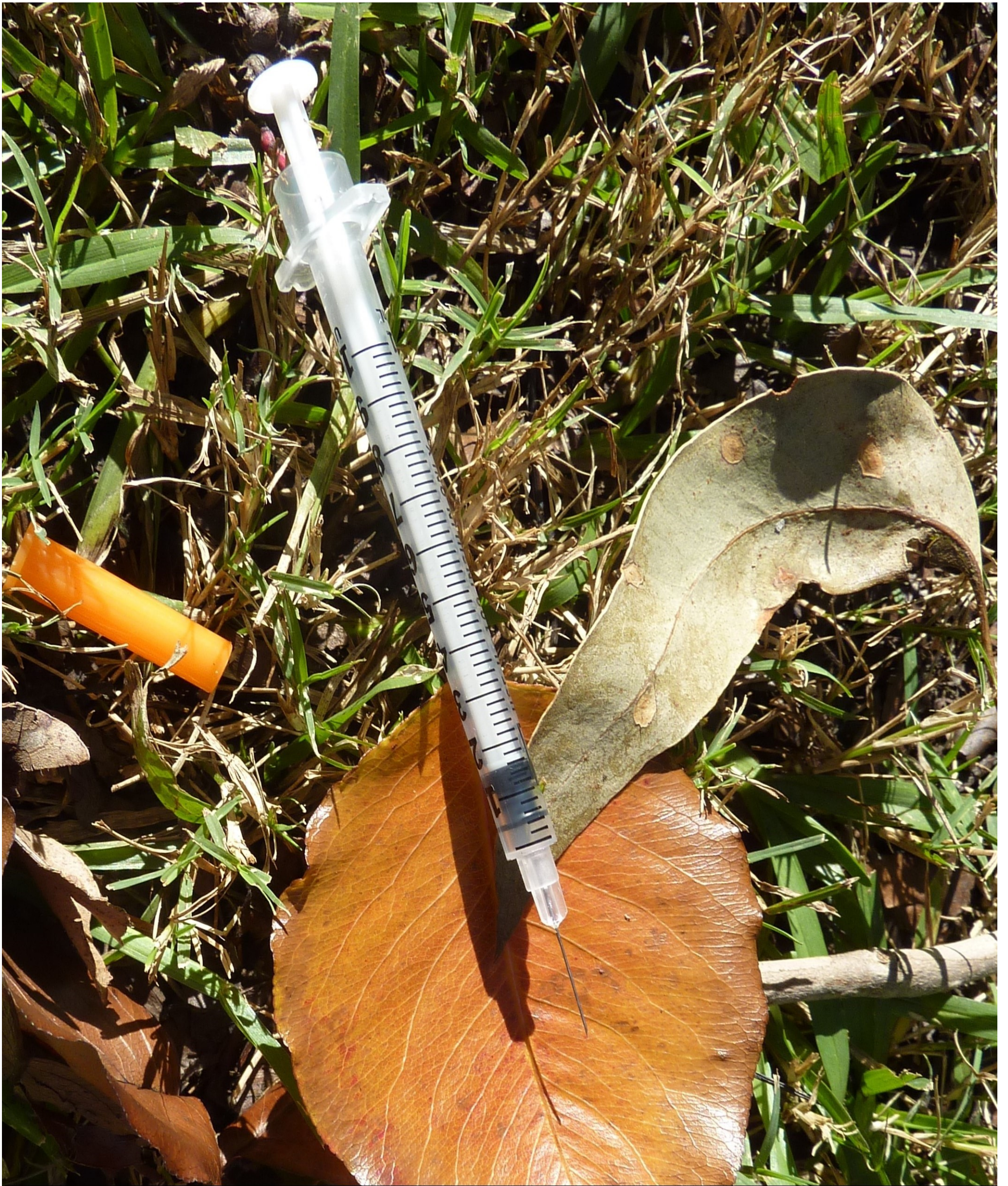

Re-using fits hurts  
...new fit every hit

Supplement: Additional file 3: — Poster 3—Re-using fits hurts. [file 12954_2015_42_MOESM3_ESM.pdf]

**ALWAYS  
use a  
new  
needle  
and  
rotate  
your  
sites**

**SAVE  
your  
vein  
for  
next  
time**

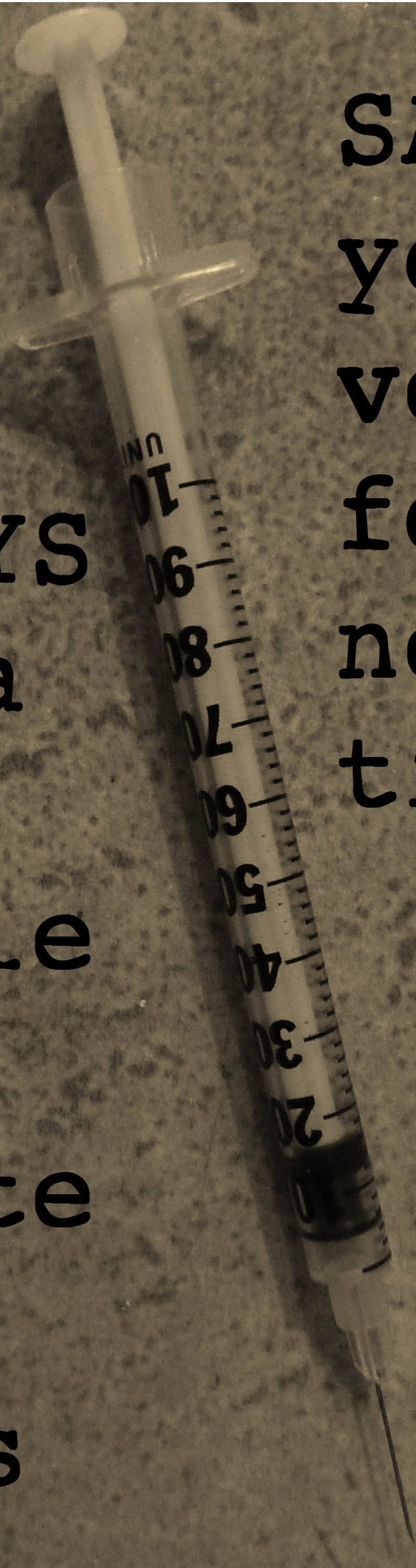

Supplement: Additional file 4: — Poster 4—Save your vein for next time. [file 12954_2015_42_MOESM4_ESM.pdf]

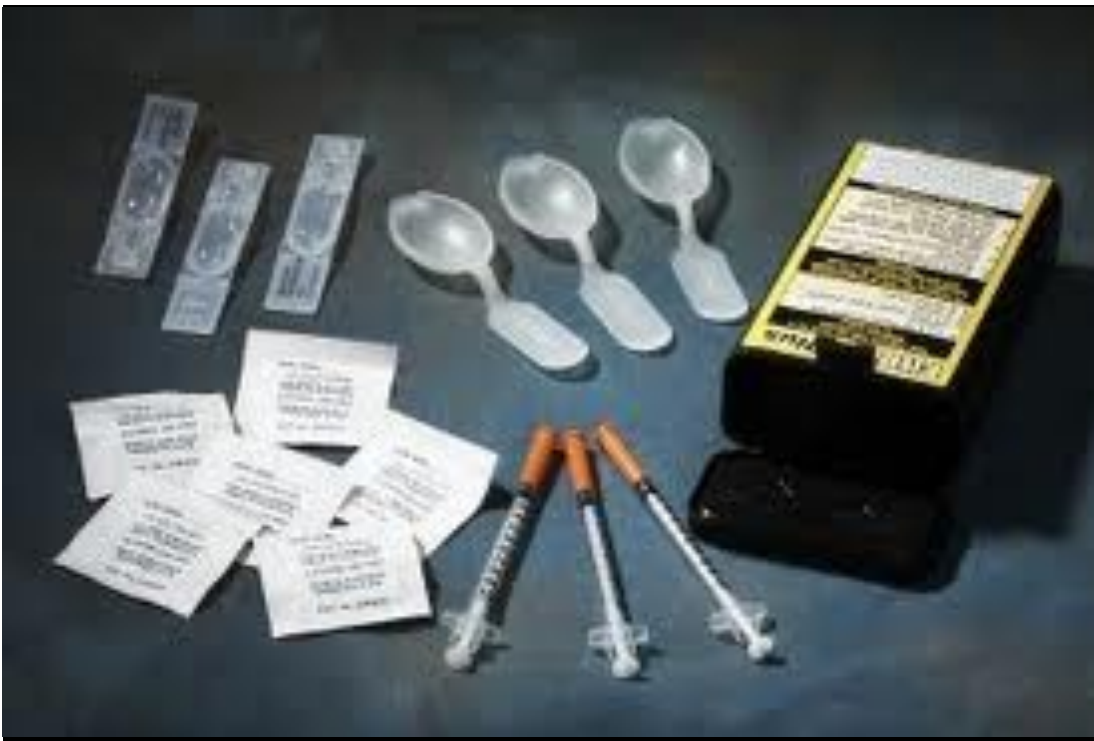

# 3

## top tips for a great hit

**1** new fit every hit

**2** shoot to the heart

**3** drink plenty of water

Supplement: Additional file 8: — Poster 8—3 top tips for a great hit. [file 12954_2015_42_MOESM8_ESM.pdf]

**blow your brains ...  
not your veins**

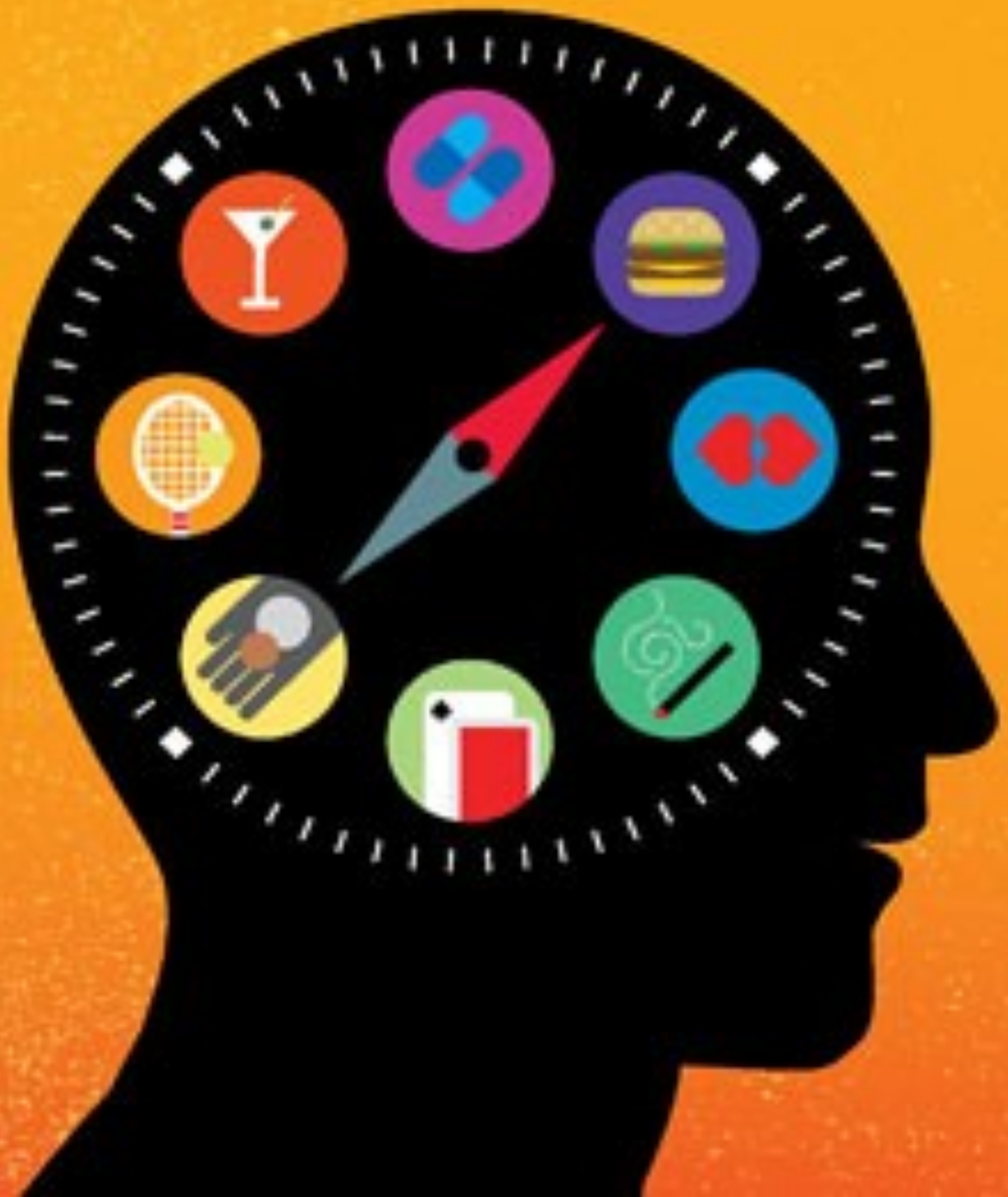

**use a new fit every hit**

Supplement: Additional file 10: — Poster 10—Blow your brains … not your veins. [file 12954_2015_42_MOESM10_ESM.pdf]

it's a fine line between  
pleasure and pain

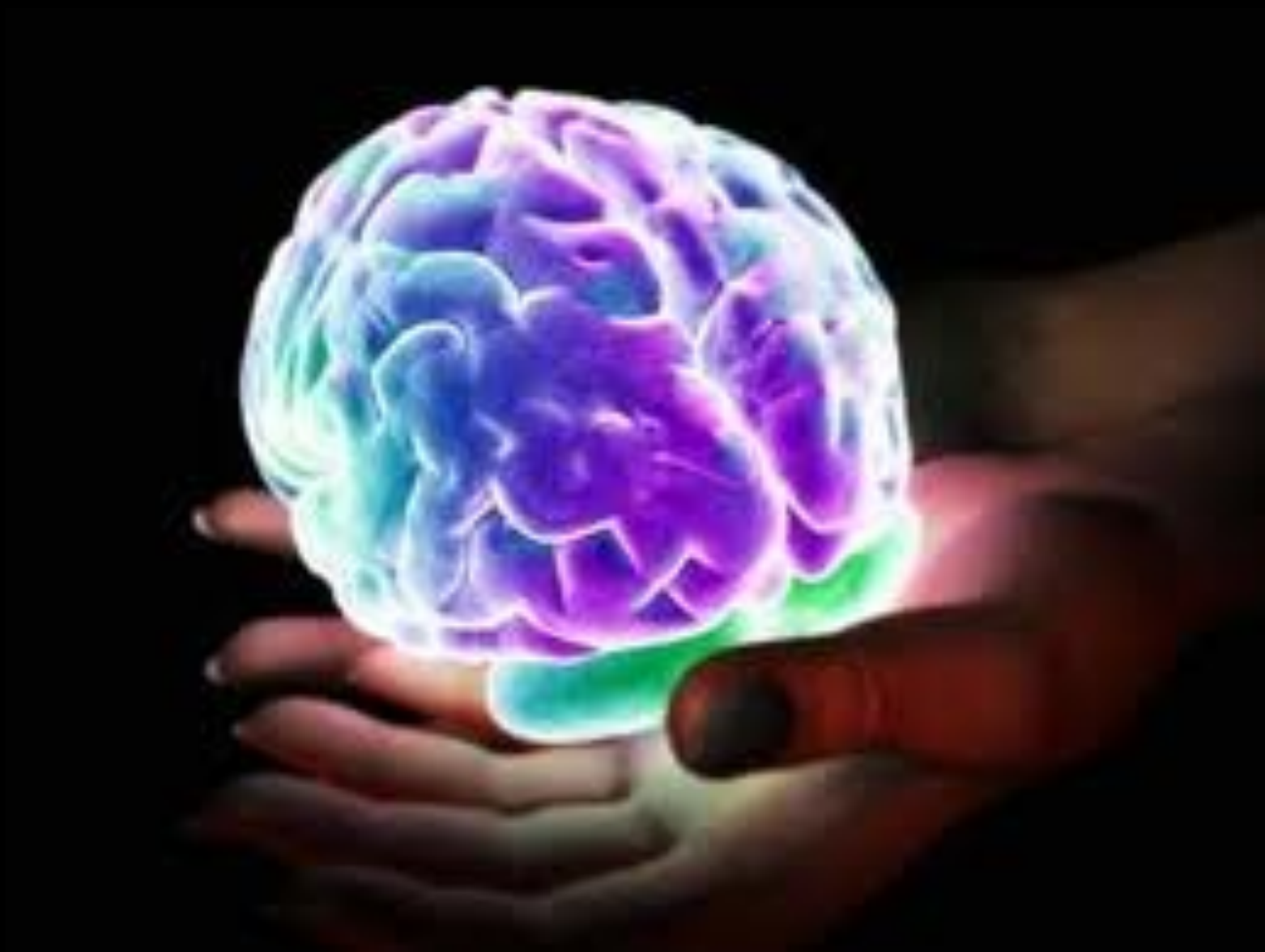

so use a pick once  
don't use it again

Supplement: Additional file 11: — Poster 11—It’s a fine line between pleasure and pain. [file 12954_2015_42_MOESM11_ESM.pdf]

new fit for a  
perfect shot

BULLSEYE

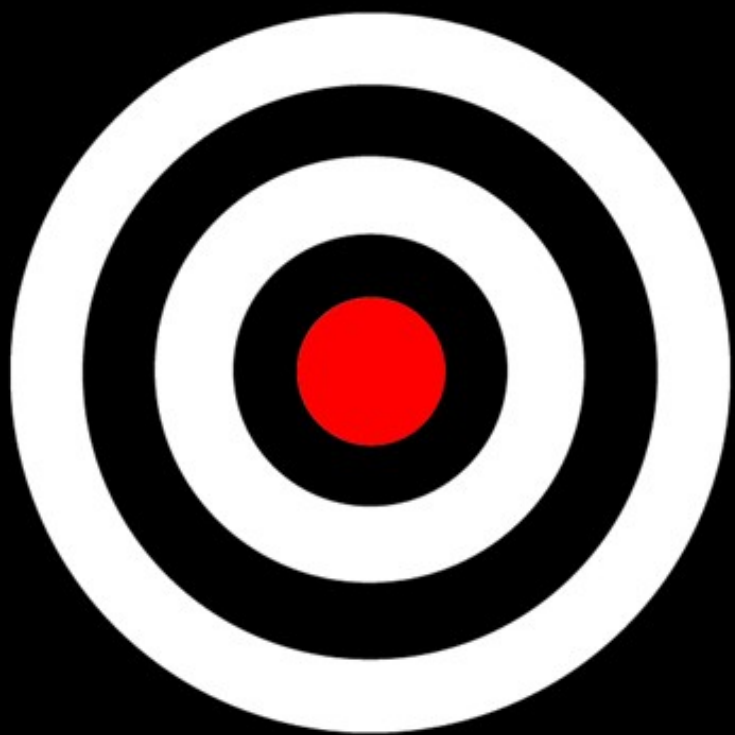

'cause blunt fits blow veins

Supplement: Additional file 12: — Poster 12—New fit for a perfect shot—bullseye. [file 12954_2015_42_MOESM12_ESM.pdf]
