# Supplementary material for: A qualitative study trialling the acceptability of new hepatitis C prevention messages for people who inject drugs: symbiotic messages, pleasure and conditional interpretations
Source: Harm Reduct J. 2015 Mar 4;12:5. doi: 10.1186/s12954-015-0042-5 (PMC4355982; doi:10.1186/s12954-015-0042-5)
Supplement: Additional file 5: — Poster 5—Some things are meant to be seen. [file 12954_2015_42_MOESM5_ESM.pdf]

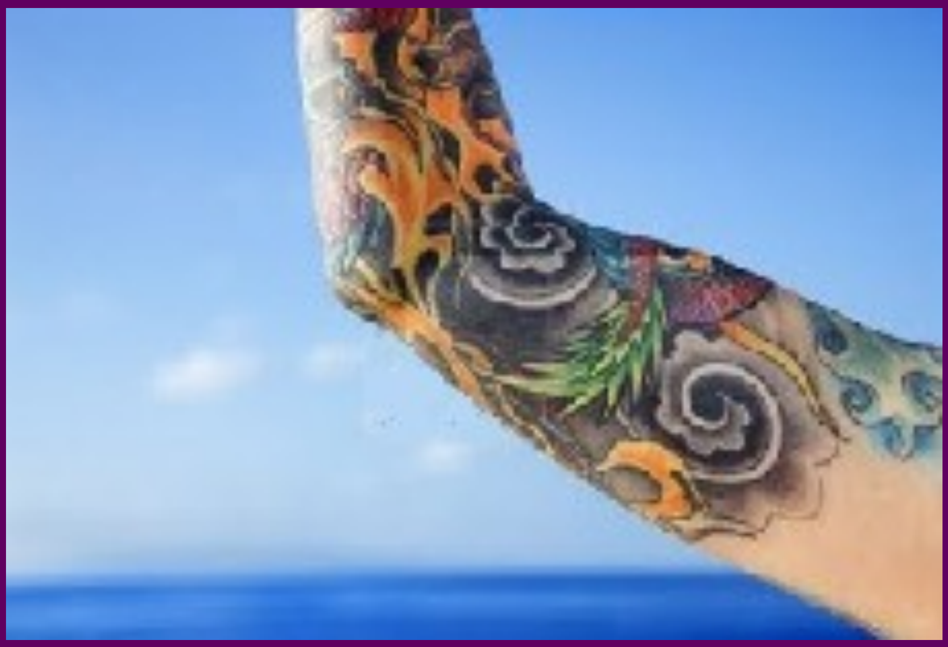

Some things are meant to be seen

...but not track marks...

avoid trackies...
use a new fit every time
and look after your veins
