# Supplementary material for: A qualitative study trialling the acceptability of new hepatitis C prevention messages for people who inject drugs: symbiotic messages, pleasure and conditional interpretations
Source: Harm Reduct J. 2015 Mar 4;12:5. doi: 10.1186/s12954-015-0042-5 (PMC4355982; doi:10.1186/s12954-015-0042-5)
Supplement: Additional file 6: — Poster 6—Prior Planning Prevents Problems. [file 12954_2015_42_MOESM6_ESM.pdf]

Prior

Planning

Prevents

Problems

P

no matter which way you look at it

time place money equipment

equipment place time money

money time equipment place

place equipment money time
