# Supplementary material for: A qualitative study trialling the acceptability of new hepatitis C prevention messages for people who inject drugs: symbiotic messages, pleasure and conditional interpretations
Source: Harm Reduct J. 2015 Mar 4;12:5. doi: 10.1186/s12954-015-0042-5 (PMC4355982; doi:10.1186/s12954-015-0042-5)
Supplement: Additional file 7: — Poster 7—These are the only trackies you want to be seen in. [file 12954_2015_42_MOESM7_ESM.pdf]

these are the only trackies you want to be seen in

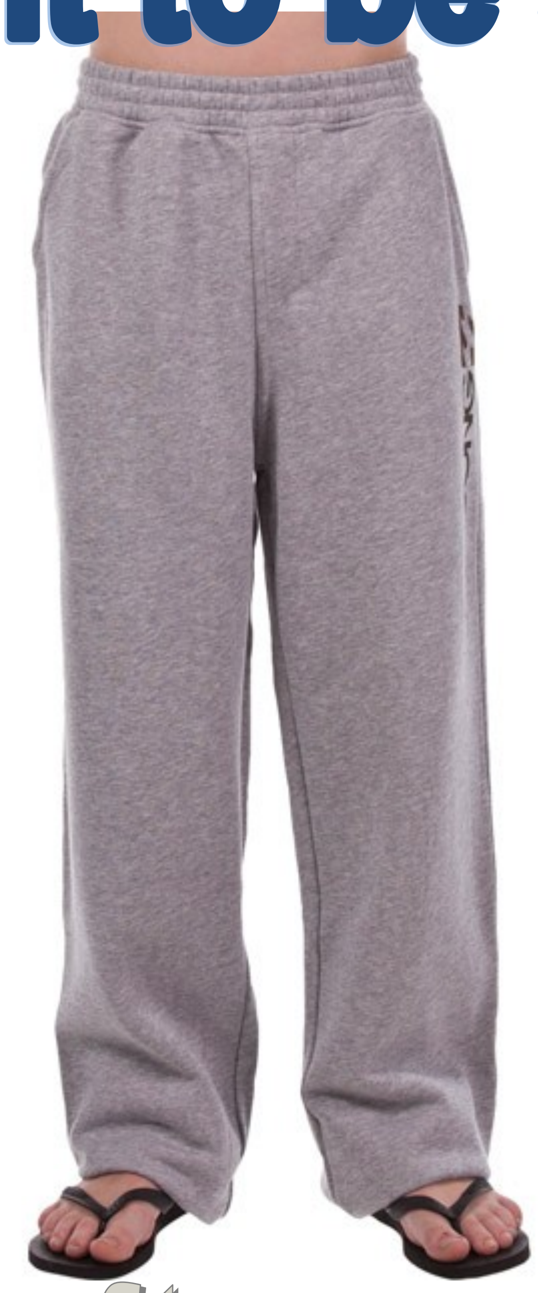

...so look after your veins ...

use a new fit

drink plenty of water

shoot to the heart

rotate your sites
